# Establishment of A Nomogram for Predicting the Prognosis of Soft Tissue Sarcoma Based on Seven Glycolysis-Related Gene Risk Score

**DOI:** 10.3389/fgene.2021.675865

**Published:** 2021-12-02

**Authors:** Yuhang Liu, Changjiang Liu, Hao Zhang, Xinzeyu Yi, Aixi Yu

**Affiliations:** Department of Trauma and Microsurgery Orthopedics, Zhongnan Hospital of Wuhan University, Wuhan, China

**Keywords:** glycolysis-related gene, prognostic model, bioinformatics analysis, soft tissue sarcoma, biomarker, nomogram

## Abstract

**Background:** Soft tissue sarcoma (STS) is a group of tumors with a low incidence and a complex type. Therefore, it is an arduous task to accurately diagnose and treat them. Glycolysis-related genes are closely related to tumor progression and metastasis. Hence, our study is dedicated to the development of risk characteristics and nomograms based on glycolysis-related genes to assess the survival possibility of patients with STS.

**Methods:** All data sets used in our research include gene expression data and clinical medical characteristics in the Genomic Data Commons Data Portal (National Cancer Institute) Soft Tissue Sarcoma (TCGA SARC) and GEO database, gene sequence data of corresponding non-diseased human tissues in the Genotype Tissue Expression (GTEx).Next, transcriptome data in TCGA SARC was analyzed as the training set to construct a glycolysis-related gene risk signature and nomogram, which were confirmed in external test set.

**Results:** We identified and verified the 7 glycolysis-related gene signature that is highly correlated with the overall survival (OS) of STS patients, which performed excellently in the evaluation of the size of AUC, and calibration curve. As well as, the results of the analysis of univariate and multivariate Cox regression demonstrated that this 7 glycolysis-related gene characteristic acts independently as an influence predictor for STS patients. Therefore, a prognostic-related nomogram combing 7 gene signature with clinical influencing features was constructed to predict OS of patients with STS in the training set that demonstrated strong predictive values for survival.

**Conclusion:** These results demonstrate that both glycolysis-related gene risk signature and nomogram were efficient prognostic indicators for patients with STS. These findings may contribute to make individualize clinical decisions on prognosis and treatment.

## Introduction

Soft tissue sarcoma (STS) accounts for 1% of adult cancer. Although its occurrence is relatively rare, STS highly heterogeneous. In the United States, approximately 13,500 new people were diagnosed with STS in 2019 (Siegel et al., 2019). There are more than 100 subtypes of STS, and the clinical characteristics of each subtype are different (Choi and Ro, 2020). In general, even if the primary tumor is removed, 25% of the patients will develop distant metastasis (Brennan et al., 2014). The ability to precisely predict the outcome on the basis of every patient’s clinical information, pathological and molecular features have attracted growing attention especially in the era of precision tumor treatment. A number of reports in literature have predicted the survival status of STS patients (Mariani et al., 2005; Cahlon et al., 2012; Callegaro et al., 2016; Gamboa et al., 2020). However, nomograms in these studies were all limited to be based on the clinical features that can only be determined after surgery; in consequence, their clinical applications might be restricted. The ideal nomograms should also include biomarkers, molecular signatures, and genomic expression to help predict survival status more accurately (Gamboa et al., 2020). With the rapid development of bioinformatics tools, multiple biomarkers for clinical diagnosis, treatment, and prognosis prediction have been identified (Wu et al., 2018; Li et al., 2019a; Long et al., 2019). The heterogeneity of the genome and the low response to traditional therapies warrant the development of effective therapeutic targets. Therefore, it is essential to determine potential precise new clinical prognostic biomarkers and therapeutic targets.

Metabolic pattern changes are one of the hallmarks of tumor cells. They tend to have a higher glycolysis efficiency, which is called the Warburg effect (Ganapathy-Kanniappan and GeschwindGeschwind, 2013). Glycolysis is essential for the development, invasion, metastasis, and drug resistance of tumor cells (Gatenby and Gillies, 2004). Moreover, previous studies have shown that targeting glycolysis-related metabolic pathways can effectively inhibit the growth of tumor cells (Abdel-Wahab et al., 2019). However, only few studies have investigated the role of glycolysis-related genes in STS. Huangyang et al. reported that the expression level of gluconeogenic isozyme fructose-1,6-bisphosphatase 2 (FBP2) was down-regulated in most subtypes of STS, and the re-expression of FBP2 significantly inhibited tumor growth (Huangyang et al., 2020). Mao et al. (2016) found that melatonin could inhibit the Warburg effect and directly inhibit the growth of leiomyosarcoma tumors. However, further studies on the mechanism of glycolysis-related genes are still necessary to develop more effective treatment for STS patients.

The Cancer Genome Atlas (TCGA) project aims to provide comprehensive transcriptome data and corresponding clinical information for various cancer patients (Jia et al., 2018). The GTEx database provides transcriptome sequencing data of 54 normal tissue samples from nearly 1,000 individuals (GTEx Consortium, 2015).

**FIGURE 1:**
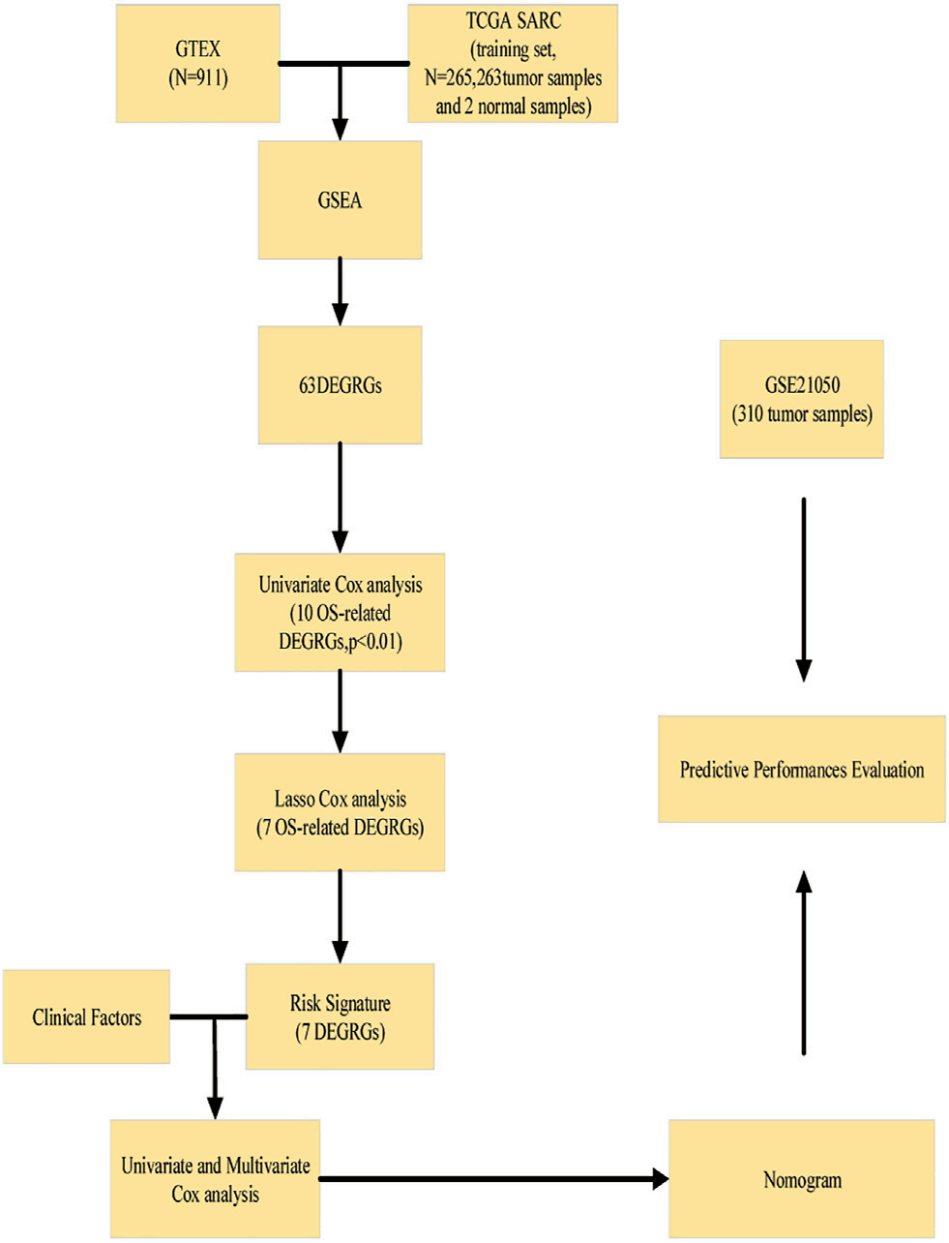
Flowchart of construction and validation of the signature and nomogram.

In our work, we comprehensively analyzed the RNA sequencing profile and clinical characteristics of the TCGA-SARC data set and the GTEx data set to screen out 7 glycolysis-related genes that are related to the prognosis of STS patients, and established a 7 gene prognostic signature. Furthermore, we constructed and verified a predictive nomogram for STS patients based on the 7-gene signature, which may aid in determining the prognostic status of STS patients and guiding tumor therapy and postoperative monitor. The workflow of our study is shown in Figure 1.

## Materials and Methods

### Data Obtainment and Preliminary Collation

We downloaded the RNA expression profiles with corresponding clinical features from the TCGA-SARC and GTEx databases at the UCSC Xena website (https://xena.ucsc.edu/). The TCGA database includes the results of large-scale sequencing of 33 human tumors and the corresponding clinical information, which helps study the molecular mechanism of tumors. Sequencing data (RNA FPKM values) of 265 samples (including 263 sarcoma samples and 2 normal tissue samples) were obtained and transformed by log_2_(FPKM+1). The GTEx database includes RNA transcriptome data of 54 normal tissue samples from healthy individuals. We obtained the RNA sequencing data (FPKM value) of corresponding muscle and adipose tissues from the GTEx database and used them as a control for additional matches. Correspondingly, the RNA sequencing data in GTEx were also converted by log_2_ (FPKM+1) for comparison with those in TCGA.

The gene expression data of GSE21050 were obtained from the GEO database (https://www.ncbi.nlm.nih.gov/geo/). The GSE21050 gene sequencing data were based on the GPL570 platform, which contains 310 sarcoma samples. The samples of TCGA-SARC were determined as the training set, while the GSE21050 dataset was identified as an external validation.

### Identification of Differentially Expressed Genes Related to Glycolysis

First, we merged the transcriptome data in TCGA-SARC and GTEx, including a total of 913 normal samples and 263 tumor samples. In order to screen out glycolysis-related genes, we performed Gene Set Enrichment Analysis (GSEA; version 4.1) based on the gene set downloaded from Molecular Signatures Database v5.1: BIOCARTA_GLYCOLYSIS_PATHWAY, GO_GLYCOLYTIC_PROCESS, HALLMARK_GLYCOLYSIS, KEGG_GLYCOLYSIS_GLUCONEOGENESIS, and REACTOME_GLYCOLYSIS. Then, the 63 differentially expressed glycolysis-related genes (DEGRGs) were identified based on the criteria of |log_2_FC|>1 and the adjusted *p* value < 0.05.

### Identification of DEGRGs Related to Overall Survival and Establishment of Prognostic Signatures

To identify the prognostic DEGRGs, the 263 sarcoma samples were analyzed as the training set, and the matched TCGA-SARC clinical features were acquired from UCSC Xena (https://xenabrowser.net/). The following data analyses were completed in R software (version 3.6.2). Firstly, the “survival” package (version 3.2.7) in R software was used to perform univariate Cox regression to analyze the 63 DEGRGs identified above. Then, DEGRGs with *p* value < 0.05 were selected to perform the least absolute shrinkage and selection operator regression (LASSO) analysis based on the “glmnet” (version 4.0.2) and “survival” (version 3.2.7) packages. LASSO is a biased estimate for processing data with multicollinearity, which identifies an optimum lambda value. Finally, a 7 DEGRGs signature related to the prognosis of STS patients was developed, and the risk score of each patient with STS was generated and calculated as follows: Risk Score = $\overset{n}{\underset{i=0}{\sum }}\beta_{i}$ * $G_{i}$ ,where $\beta_{i}\;$ is defined as the coefficient of gene   i of the LASSO analysis; $\;G_{i}$ presents the expression level of each gene. Based on this gene signature, STS patients in the training set were divided into high-risk and low-risk groups on account of the critical value (i.e., the median risk score). A total of 309 samples in GSE21050 (survival data missing in 1 sample) were identified as the test set. To assess the accuracy of results, we analyzed the data in the test set at the same level. To assess the availability of the signature, we conducted overall survival analysis to evaluate the overall survival differences in the high-risk and low-risk patients. Receiver operating characteristic (ROC) curves were obtained using the “survivalROC” package (version 1.0.2). The ROC curves at 3 and 5 years were also generated to evaluate the credibility and accuracy of the risk signature.

### Analysis of the Clinical Characteristics Associated With Prognosis

Univariate Cox regression and multivariate Cox regression analyses were conducted to evaluate the influences of clinical features on the prognosis of patients. We eliminated the samples with incomplete clinical information in the training set, and those with complete information were selected for the next processing. The Cox regression analyses included the following factors: age, margin, metastasis, depth, ethnicity, gender, race, diagnoses, and the risk score identified above. Multiple ROC curves were constructed using the “survivalROC” package in R software.

### Establishment of Prognosis-Related Nomogram and Validation

We combined the risk signature with the factors identified above to build a nomogram for prognostic prediction of sarcoma patients in the training set, using the “rms” package (version 6.0.1) in R software. Meanwhile, the 1, 3, and 5 years calibration curves were created to assess the consistency between the realistic results and the results demonstrated by the nomogram in the training set.

## Results

### Patient Characteristics and Transcriptome Expression Level

TCGA-SARC RNA sequence expression data and the corresponding clinical information were obtained from UCSC Xena. There were 265 sequence profiles in TCGA-SARC, with 263 tumor samples and 2 normal samples. The 263 STS samples were contained, including 105 leiomyosarcomas (LMS), 56 dedifferentiated liposarcomas (DL), 34 undifferentiated sarcomas (US), 25 myxofibrosarcomas (MF), 12 malignant fibrous histiocytomas (MFH), 10 malignant peripheral nerve sheath tumors (MPNST), 10 synovial sarcomas (SS), 3 myxoid leiomyosarcomas, 3 giant cell sarcomas, 2 pleomorphic liposarcomas, and 3 other sarcoma samples. The matched normal samples obtained from the GTEx database included 911 normal tissue samples (396 muscle and 515 adipose samples). Finally, we identified 263 tumor samples and 913 healthy tissue samples. Eventually, the expression levels of 19,532mRNAs were identified.

### Screening of DEGRGs

**FIGURE 2:**
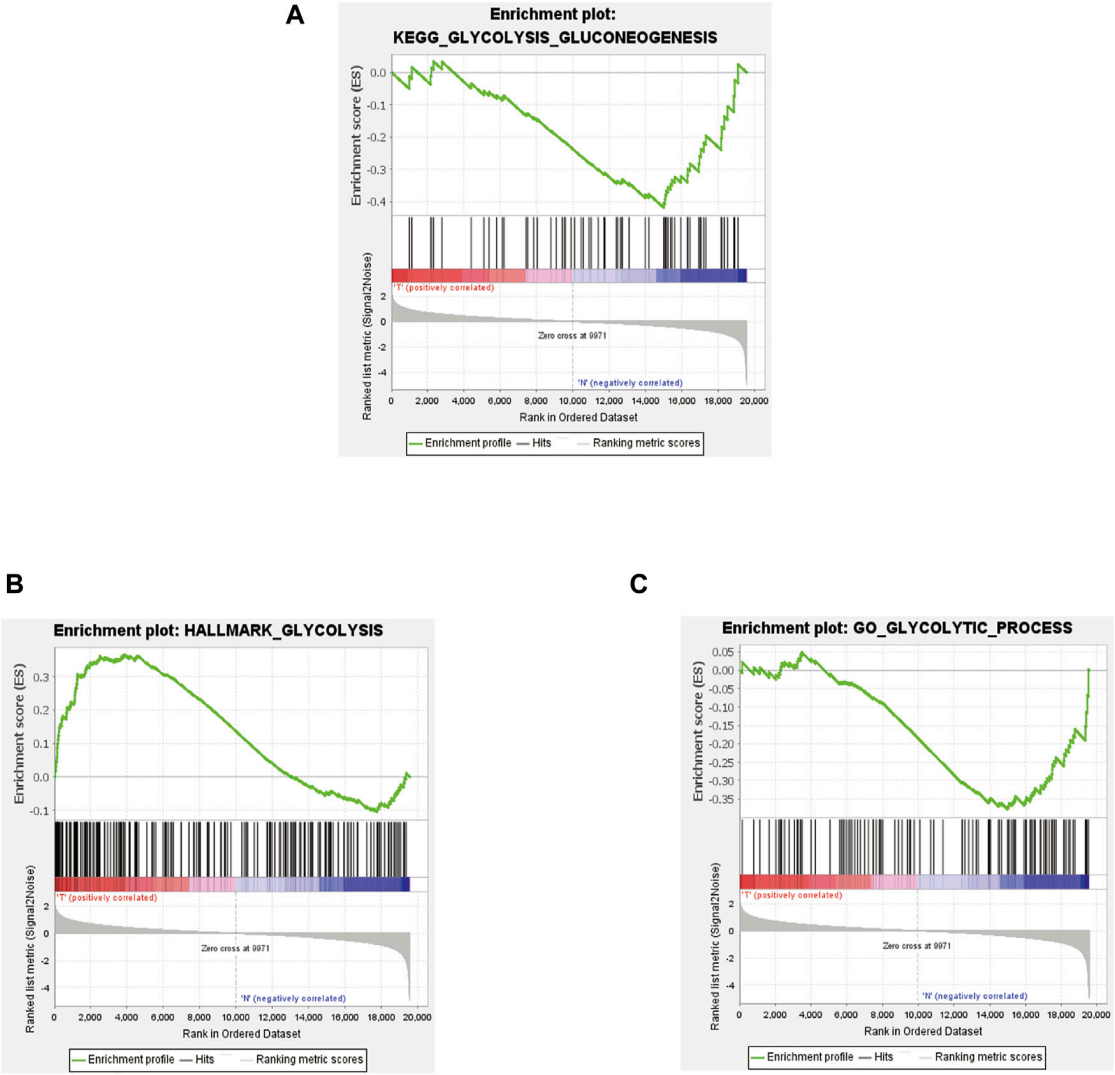
Results of GSEA based on glycolysis-related gene sets. KEGG_GLYCOLYSIS_GLUCONEOGENESIS **(A)**, HALLMARK_GLYCOLYSIS **(B)**, GO_GLYCOLYTIC_PROCESS **(C)**.

Five gene sets related to glycolysis were downloaded from the Molecular Signatures Database v5.1, and a total of 326 gene expression data were generated. In order to analyze the difference levels of 5 glycolysis-related gene sets in STS and normal samples, we performed GSEA analysis (version 4.1). The analysis results of DEGRGs are shown in Figure 2.

**FIGURE 3:**
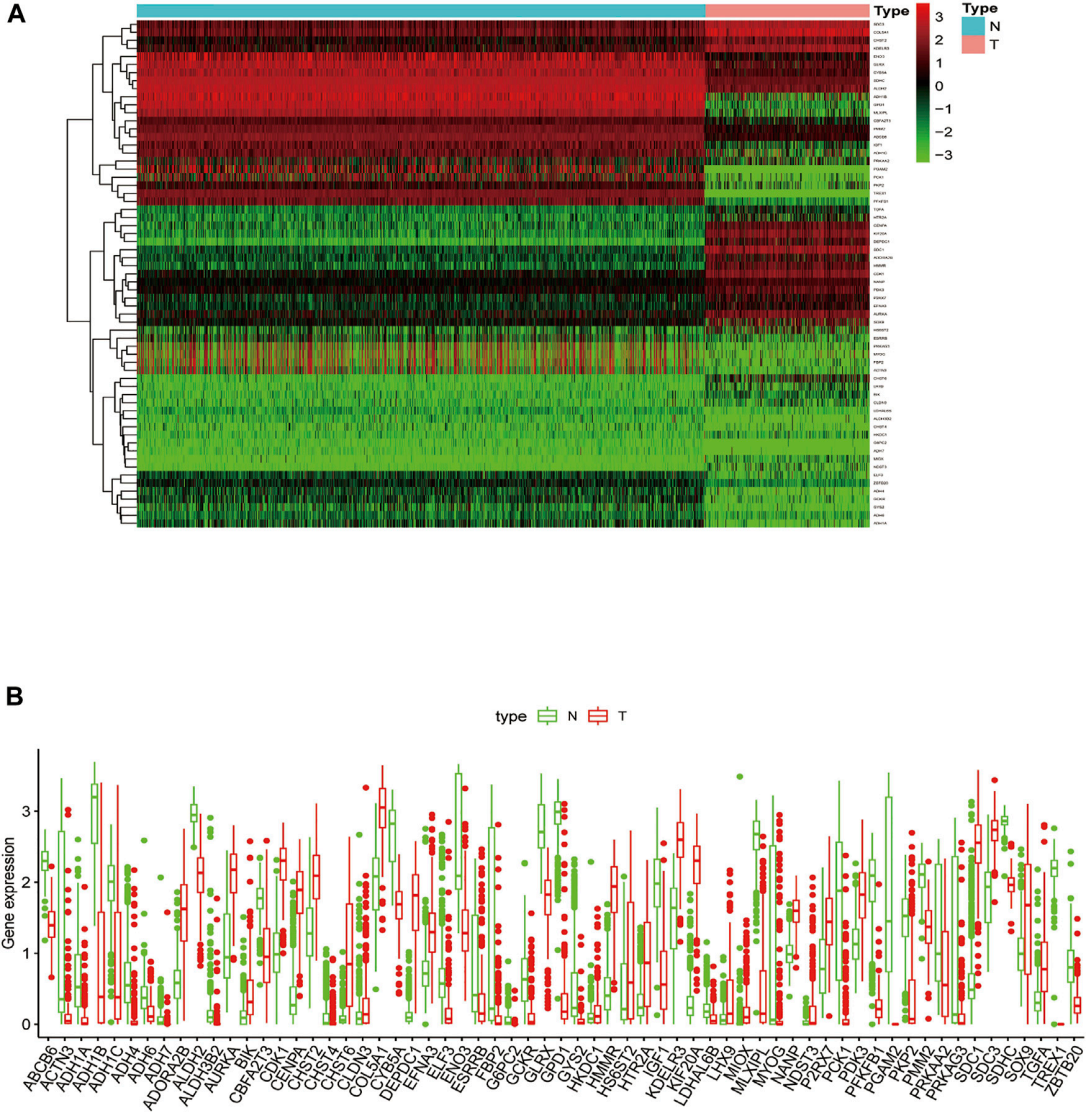
Differently expressed glycolysis-related genes (DEGRGs) between sarcoma samples and normal tissues. The screening criteria was based on the |log_2_FC| ≥1 and p<0.05. **(A)** Heatmap of DEGRGs **(B)** Box plot of DEGRGs. FC, fold change. Color images are available online.

Next, we obtained the expression levels of a total of 326 glycolysis-related genes from these 5 gene sets in the training set. Based on the criteria of |log_2_FC|≥1 and adjusted *p* < 0.05, 63 DEGRGs were identified, including 34 down-regulated and 29 up-regulated genes. The heatmap (Figure 3A) and boxplot (Figure 3B) of these 63 DEGRGs were generated in R software. The glycolysis-related gene expression matrix are shown in the Supplementary Materials.

### Risk Signature Construction

**FIGURE 4:**
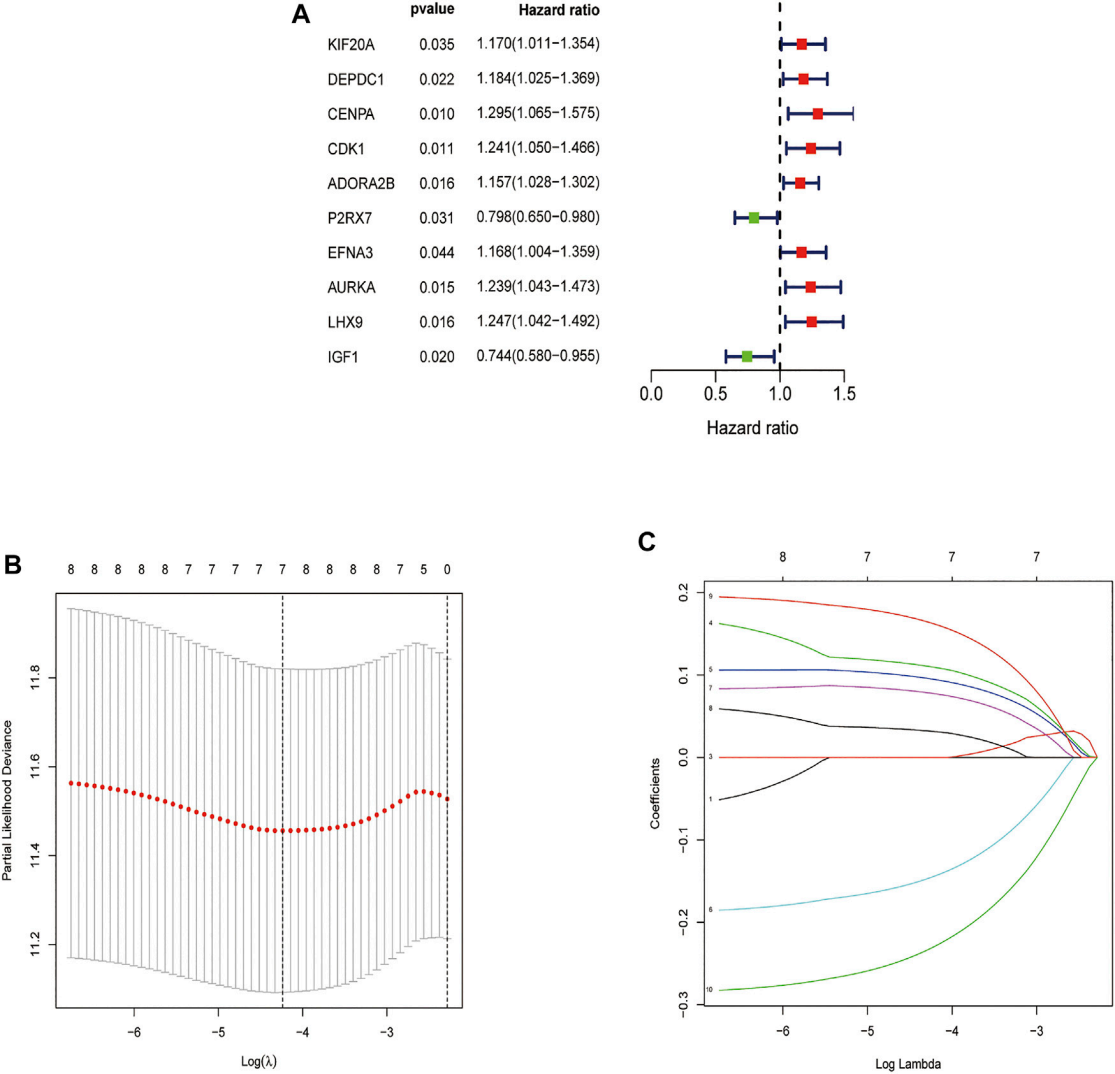
Feature selection using the Univariate Cox analysis and Lasso regression model. **(A)** Forest map of DEGRGs associated with STS survival, univariate Cox regression, *p* < 0.01. **(B)** LASSO coefficient spectrum of 7 DEGRGs. **(C)** On account of 1000 cross‐validation for tuning parameter selection via LASSO.

In order to determine the overall survival-related DEGRGs, univariate Cox regression analysis was applied to analyze the above identified 63 DEGRGs in the training set, and 10 genes were selected (Figure 4A). Then, using the “glmnet” package, DEGRGs with *p* < 0.01 were further used for the LASSO regression analysis to establish a gene signature (Figures 4B,C). The risk score of every patient was acquired by multiplying the gene level (X) with the regression coefficient (ɑ). Finally, 7 DEGRGs highly related to prognosis were utilized to build a prognostic-related model: risk score = (X _CDK1_ * 0.1079) + (X _ADORA2B_ * 0.0936) + (X _P2RX7_ * -0.1412) + (X _EFNA3_ * 0.0764) + (X _AURKA_ * 0.0311) + (X_LHX9_ * 0.1598) + (X _IGF1_ * −0.2258).

**FIGURE 5:**
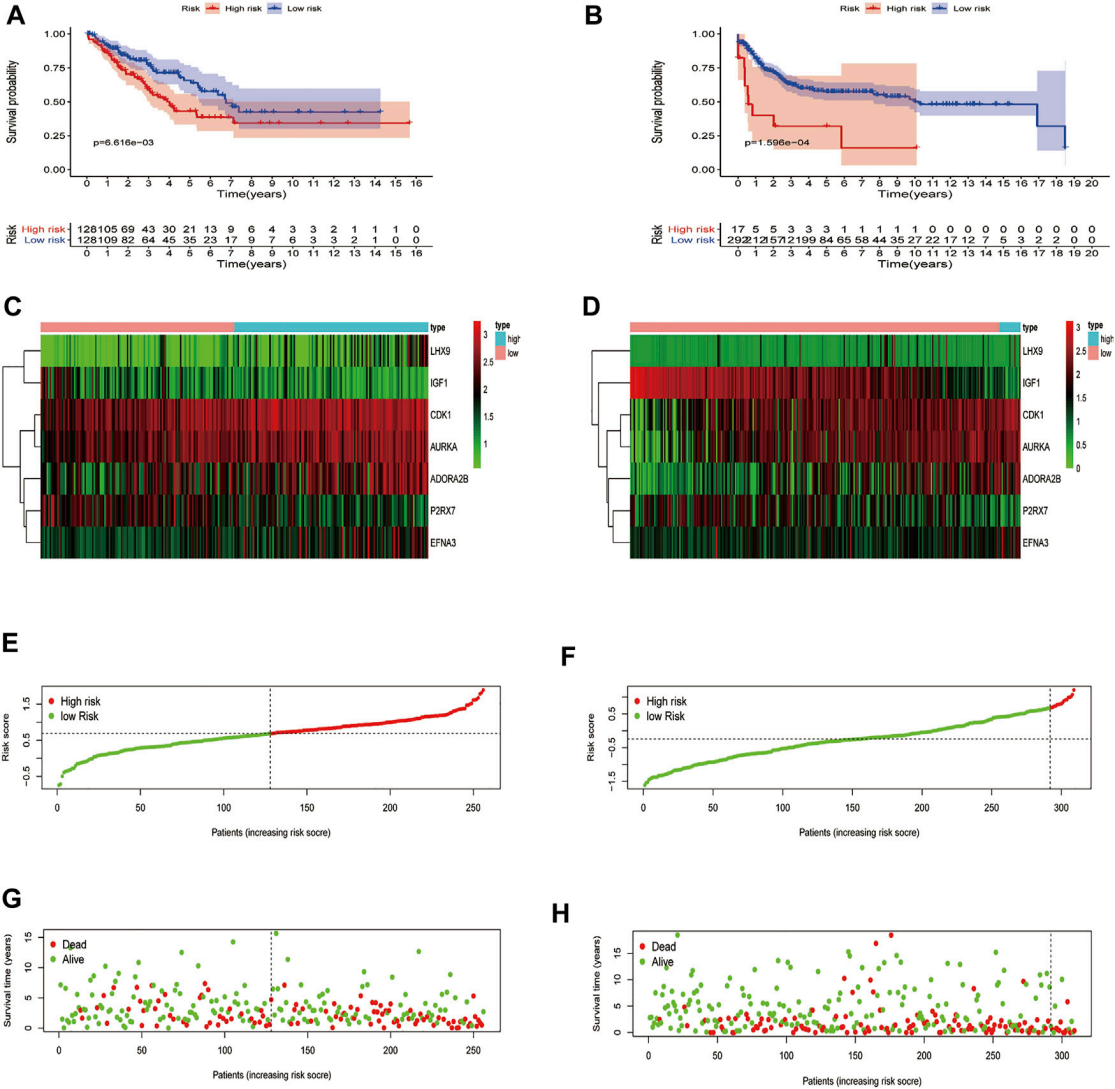
Risk signature development. Survival analysis of the training **(A)**, test **(B)**. The upper part demonstrates the KM(Kaplan-Meier) curves for the high and low risk groups. The number shows the living patients with time in the two groups. Differences in gene expression level between two groups, risk distribution of per samples, and relationship between survival status and survival times, training set **(C,E,G)**, test set **(D,F,H)**. The dark line shows the cut-off point dividing the two groups.

**FIGURE 6:**
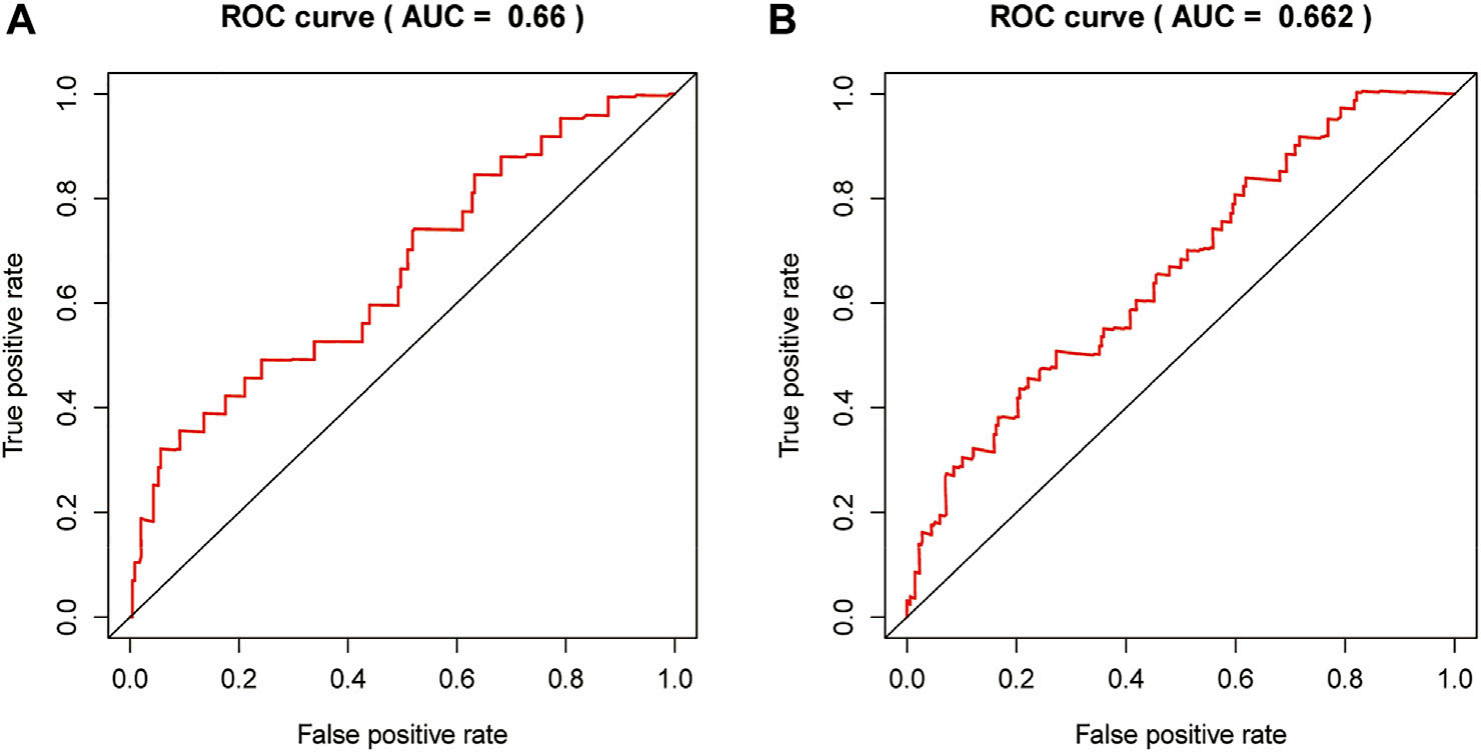
Receiver operating characteristic (ROC) of 7 DEGRGs model in the training (I), test (J).

On account of the median risk score, the patients with STS in the training set were divided into high-risk and low-risk groups. Expression heatmaps, risk distribution plots, and survival status profiles of the 7 identified DEGRGs were constructed, and the survival difference between the two groups in training set (Figures 5A,C,E,G). Similar differences were also observed in the test group, which verified the prognostic model (Figures 5B,D,F,H). As shown in Figures 6A,B, the characteristics of the 7 DEGRGs can satisfactorily predict the survival status of STS patients, with AUC = 0.66 (test set: 0.662).

### Evaluation of the DEGRGs Signature

**FIGURE 7:**
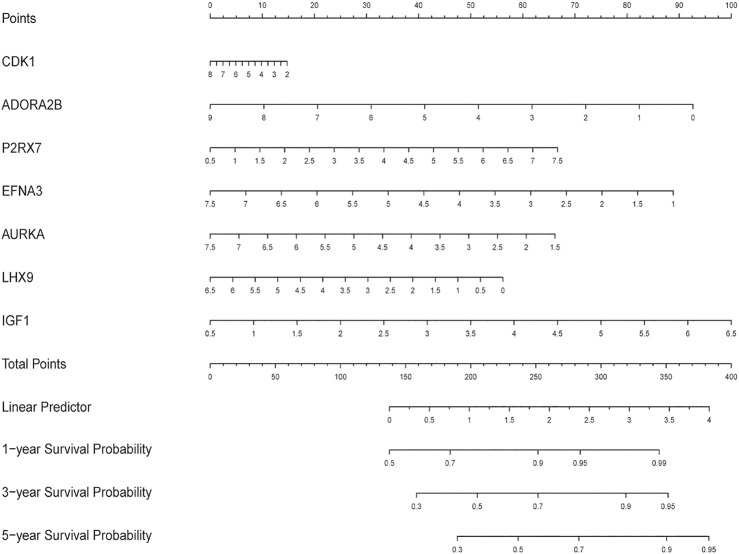
The nomogram predicting the survival status of STS patients on the base of the expression level of 7 DEGRGs.

**FIGURE 8:**
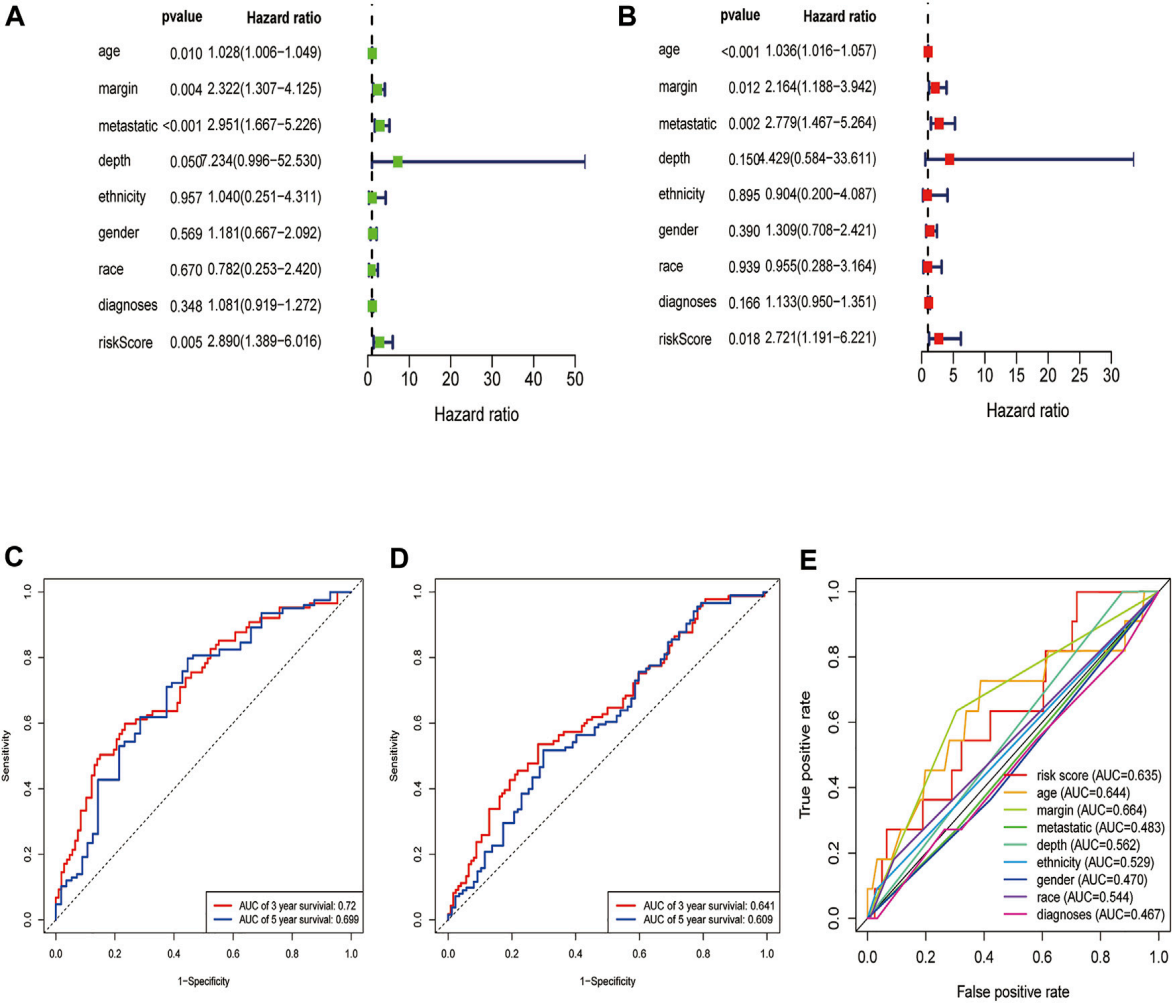
Evaluation of the DEGRGs signature. The Result of univariate and multivariate Cox analyses **(A,B)**. The AUC curves to predict the survival status of STS patients at the 3- and 5-year survival time in train set **(C)**, test set **(D)**.The multi ROC curves of risk model and other clinical characteristic.

First, we constructed a nomogram according to the expressions of the 7 DEGRGs to predict the prognosis status of STS patients (Figure 7). The results from univariate and multivariate independent prognostic analyses showed that the risk characteristics of the 7 DEGRGs were significantly (*p* < 0.05) related to the survival status of STS patients (Figures 8A,B). Analysis of multiple ROC curves showed that risk score signature had the largest AUC area (Figure 8E). The AUC size represents the prognostic efficiency of the 7-DEGRGs model. The larger the area, the better the predictive effect on patient’s prognosis. In addition, based on the “timeROC” package (version 0.4) in R software, curves were plotted to evaluate the predictive value (Figures 8C,D). Our results showed that the 7 DEGRGs prognostic model could predicted both 3-year survival rate (AUC = 0.72) and 5-year survival rate (AUC = 0.699). These results demonstrated the excellent accuracy and sensitivity of the model.

### Construction and Evaluation of a Nomogram Incorporating the DEGRGs Signature With Clinical Factors

**FIGURE 9:**
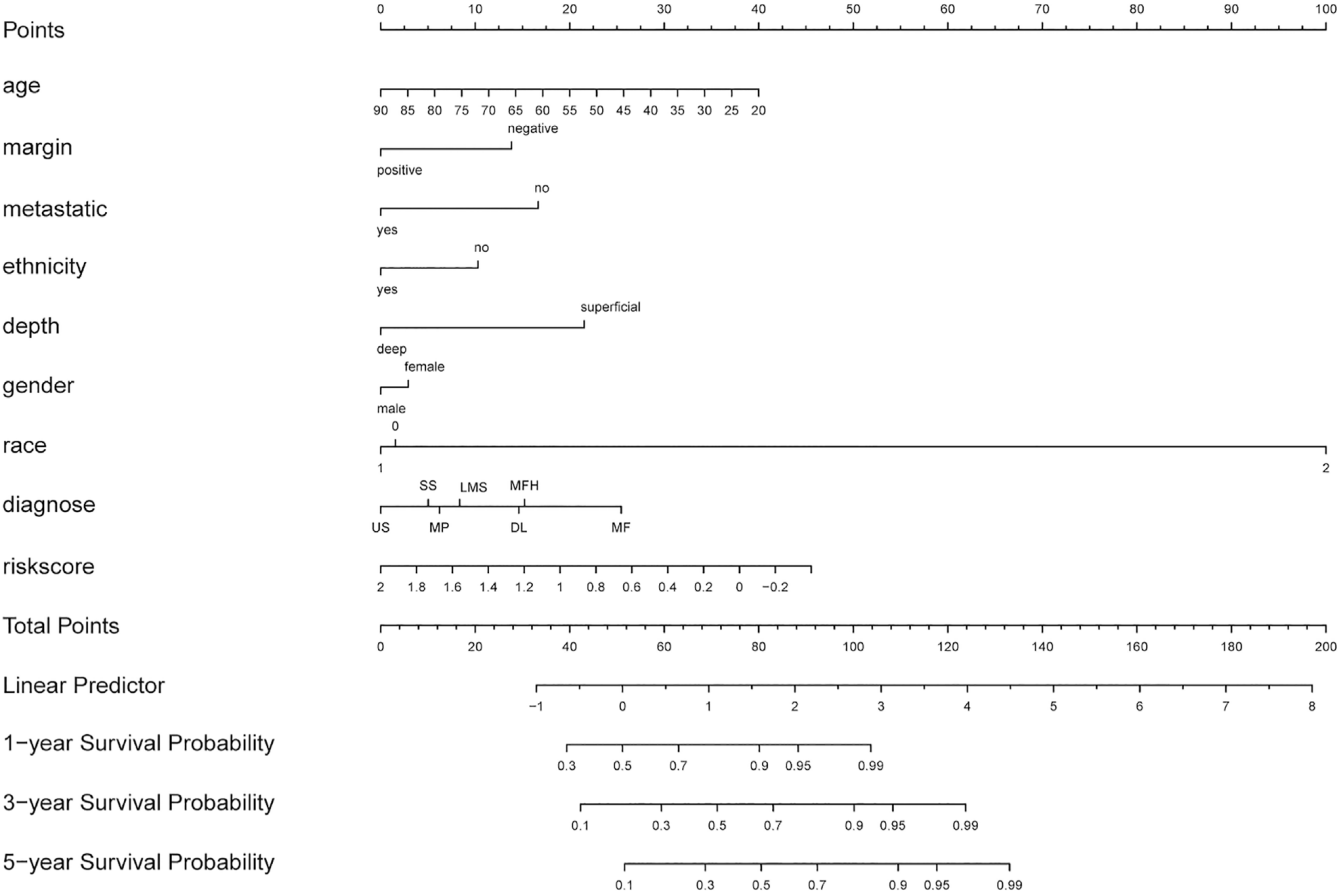
Nomogram combing risk score and clinical factors for prediction of the individualized survival probability of STS patients. In the “diagnose” row, LMS (leiomyosarcomas), DL ( dedifferentiated liposarcomas), US( undifferentiated sarcomas), MF(25 myxofibrosarcomas), MFH(malignant fibrous histiocytomas), MPNST (malignant peripheral nerve sheath tumors), SS( synovial sarcomas).

**FIGURE 10:**
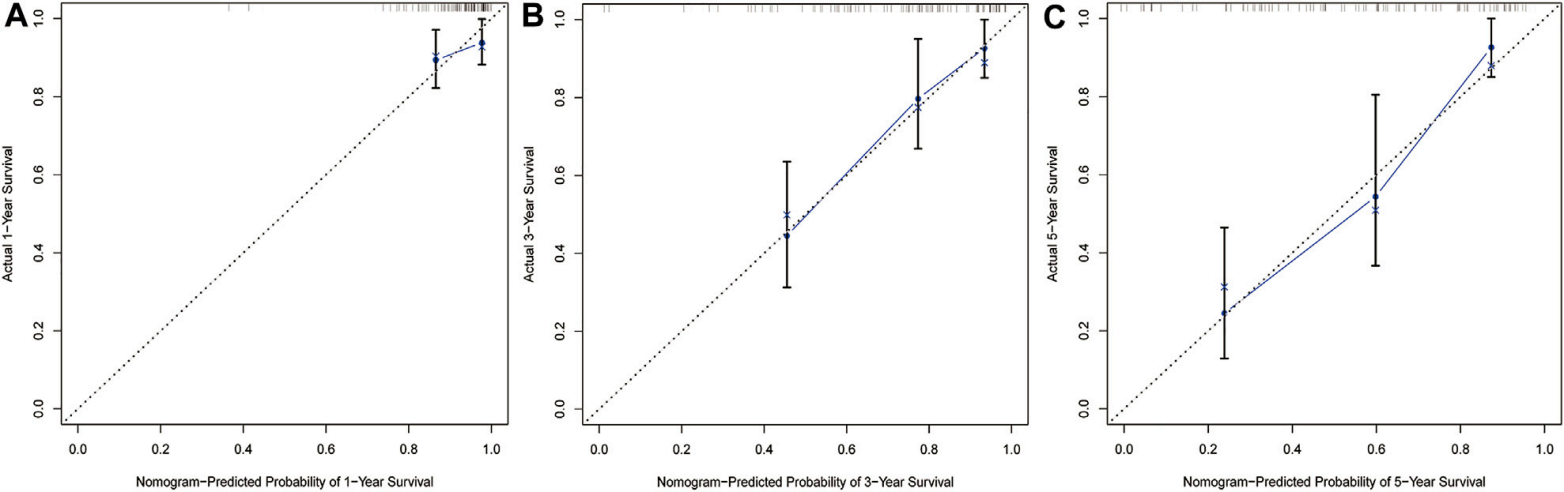
Calibration curves of nomogram in train set. The curve shows the agreement between nomogram -predicted and observed 1**(A)**-,3**(B)**-and 5**(C)**-year survival outcomes of STS patient. The closer the blue line in the figure is to the 45-degree dotted line, the better the prediction performance of the nomogram.

Based on multiple Cox regression, we constructed a prognostic nomogram to predict 1-year, 3-year, and 5-year survival possibility (Figure 9). Furthermore, calibration plots of the 1-year, 3-year, and 5-year survival prediction were used to assess the predictive ability of the nomogram, as shown in Figures 10A–C. The calibration curve showed that the nomogram had a high consistency between the actual and prediction results of survival state in the training set.

## Discussion

Glycolysis-related genes have been revealed to play an important role in the occurrence and development of tumors (Yu et al., 2017; Yang et al., 2020). YAP1 affects the glycolytic metabolism of undifferentiated pleomorphic sarcoma through the NF-κB pathway (Rivera-Reyes et al., 2018). Lactate dehydrogenase inhibitors reduce the production of lactic acid and inhibit glycolysis, thereby inhibiting the proliferation of A673 sarcoma cells (Rai et al., 2017). Phosphoglycerate dehydrogenase (PHGDH) is highly expressed in Ewing’s sarcoma and is associated with poor patient survival. PHGDH knockdown or *in vitro* pharmacological inhibition lead to decreased cell proliferation and cell death (Tanner et al., 2017). These studies indicate that glycolysis-related genes may play an important role in STS.

STS is a rare cancer, including more than 100 subtypes, with different pathological characteristics, molecular changes, and various prognosis of the patients. The efficient diagnosis and effective treatment of STS is difficult on account of the rarity and complex subtypes (Meyer and Seetharam, 2019). With the availability of more gene databases, novel analytic tools can be developed to explore biomarkers for rare tumors (e.g., STS) from existing data (van IJzendoorn et al., 2019). With the development of bioinformatics, a growing number of studies have proved that processing gene databases is an effective method to assess the transcriptome characteristics associated with prognosis, which can help identify new serum biomarkers for clinical diagnosis, prognosis prediction, as well as postoperative treatment (Ouyang et al., 2019).

Cancer cells usually have more vigorous metabolism than normal cells, which is characterized by aerobic glycolysis and anabolic cycles to support tumor metastasis and proliferation (Ancey et al., 2018; Orang et al., 2019). In recent years, an increased number of tumor-related studies have focused on investigating the glycolytic process (Abbaszadeh et al., 2020). According to these studies, multiple genes and pathways related to glycolysis have been discovered. Analogs and blockers of these genes have also been developed, involving a variety of molecules, chemical drugs, and nano-drugs (AkinsAkins et al., 2018). Several studies have also reported the mechanism of glycolysis in STS. For example, Duan et al. found that glycolysis inhibitor 2-deoxyglucose can induce alveolar rhabdomyosarcoma cell apoptosis by regulating the expression level of Noxa (Ramírez-Peinado et al., 2011).

We were committed to identifying potential glycolysis-related gene biomarkers for assessing the risk and prognosis of STS patients. In this study, we screened out 7 glycolysis-related genes and established a prognostic nomogram by combined the model with several clinical features, which was effective in predicting STS. Through the joint analysis of the TCGA and GTEx databases, 63 DEGRGs were identified, which might serve as potential biomarkers for STS. Univariate Cox regression analyses were conducted and filtered out 10 prognosis-related DEGRGs. Subsequently, the LASSO regression was performed to further analyze these 10 genes in the training set, based on which the 7 GEGRGs (CDK1, ADORA2B, P2RX7, EFNA3, AURKA, LHX9, andIGF1) model was finally established.

The functions of the identified 7 DEGRGs have been previously reported. For instance, Menon et al. demonstrated that CDK1 was up-regulated in melanoma cells and interacted with Sox2 to promote the proliferation of melanoma (Ravindran Menon et al., 2018). Desmet et al. found that blocking ADORA2B inhibited the invasive activity of breast cancer cells and reduced their ability to metastasize (Wilkat et al., 2020). ADORA2B knockdown reduced tumor vascularization and thus inhibited the growth of head and neck squamous cell carcinomas (Desmet et al., 2013). Furthermore, Wang et al. proved that P2RX7 was overexpressed in gastric cancer tissues, promoting tumor proliferation through ERK1/2 pathway and Akt pathway, which was also correlated with poor prognosis (Lili et al., 2019). Wang et al. reported that cathelicidin inhibited colon cancer metastasis through a P2RX7-dependent pathway (Wang et al., 2020). EFNA3, as an Eph receptor ligand, affected the migration and proliferation of human umbilical cord endothelial cells through the PI3K/AKT pathway (Cheng et al., 2019). Chen et al. found that AURKA directly promoted the Warburg effect by phosphorylating lactate dehydrogenase B (LDHB), thereby promoting tumor growth (Li et al., 2019b). It has also been confirmed that the expression level of LHX9 was significantly up-regulated in osteosarcoma, and inhibiting LHX9 reduced the ability of cell growth and invasion (Li et al., 2019c). Li et al. proved that the levels of IGF-1 and IGF-1R in osteosarcoma were elevated, and their overexpression promoted the invasion and resistance of osteosarcoma cells (Yu et al., 2020).

Recently, with the development of bioinformatics tools, multiple glycolysis-related gene models have been developed to assess the survival status of cancer patients (Cai et al., 2020). To our knowledge, our study is the first to screen out DEGRGs by analyzing data from the public TCGA database to predict the survival status of STS patients. Moreover, based on these 7 DEGRGs, through combining the risk score and clinical characteristics, we constructed a nomogram to assess the prognosis of STS patients. We found that glycolysis-related genes and STS prognosis were closely correlated, which may provide us with a novel strategy for the treatment of STS.

This work has some limitations. First of all, the number of STS samples in TCGA-SARC data set was relatively small, and that of normal samples was insufficient though GTEx database was also involved. Second, several important clinical features (e.g., tumor stage) of the patients in the TCGA database were not sufficiently detailed, which may affect the treatment and prognosis of STS patients. Finally, more independent external queues need to be analyzed on the basis of our model to ensure the predictive performance of the nomogram.

## Conclusion

By using the high-throughput sequencing data in the TCGA database, we performed a variety of high-dimensional regression analyses (LASSO and Cox regression models) to identify the prognostic DEGRG markers for STS patients. The 7 gene prognostic signature is an effective predictor of STS. Through combining the 7 DEGRGs and clinical characteristics of STS patients, we established a prognostic nomogram that has superior efficacy in STS risk and patient survival prediction. The significant effectiveness of this model may be helpful for decision-making in clinical treatment, and further study is warranted to reveal the biological and molecular roles of these DEGRGs in STS.

## Data Availability

The datasets presented in this study can be found in online repositories. The names of the repository/repositories and accession number(s) can be found in the article/Supplementary Material.
